# Impact of pulmonary artery pressure on recurrence after catheter ablation in patients with atrial fibrillation

**DOI:** 10.3389/fcvm.2023.1187774

**Published:** 2023-09-04

**Authors:** Yun Young Choi, Jong-Il Choi, Joo Hee Jeong, Hyoung Seok Lee, Yun Gi Kim, Mi-Na Kim, Seung-Young Roh, Jaemin Shim, Jin Seok Kim, Seong-Mi Park, Young-Hoon Kim

**Affiliations:** Division of Cardiology, Korea University College of Medicine and Korea University Medical Center, Seoul, Republic of Korea

**Keywords:** atrial fibrillation, radiofrequency catheter ablation, pulmonary atrial pressure, recurrence, atrial remodeling

## Abstract

**Background:**

The pulmonary veins play a major role in the pathogenesis of atrial fibrillation (AF) and may be affected by cardiac remodeling due to pulmonary vascular dysfunction. It remains to be determined whether pulmonary artery pressure (PAP) is associated with the recurrence of AF after radiofrequency catheter ablation (RFCA).

**Methods:**

Consecutive patients with paroxysmal and persistent AF who underwent RFCA, including wide circumferential pulmonary vein isolation, were analyzed. Systolic PAP was measured using transthoracic echocardiography, and clinical outcomes were compared between patients with PAP <35 mmHg and those with PAP ≥35 mmHg.

**Results:**

Among 2,379 patients (mean age 56.7 ± 10.6 years, 77% men), 1,893 (79.6%) had PAP <35 mmHg and 486 (20.4%) had PAP ≥35 mmHg. During the median follow-up of 25.4 months, in patients with paroxysmal AF (*n* = 1,294), the recurrence rate was significantly greater in the PAP ≥35 mmHg group than in the PAP <35 mmHg group (35.1% vs. 23.8%, log-rank *p *= 0.008). However, in patients with persistent AF (*n* = 1,085), the recurrence rate was not significantly different between the two groups (52.2% vs. 49.7%, log-rank *p *= 0.409). Multivariate analysis using Cox regression showed that PAP ≥35 mmHg was significantly associated with clinical recurrence (hazard ratio 1.19, 95% confidence interval 1.02–1.40, *p *= 0.027).

**Conclusion:**

This study showed that a higher PAP was associated with an increased risk of recurrence after RFCA in patients with paroxysmal AF, suggesting a mechanism by which a pulmonary vascular pathology may cause impairment of the pulmonary veins and remodeling of the left atrium.

## Highlights

• This is the first study on the association between pulmonary artery pressure (PAP) and recurrence after radiofrequency catheter ablation (RFCA) in patients with atrial fibrillation (AF).
• Higher PAP measured by echocardiography was associated with an increased risk of recurrence after RFCA in patients with paroxysmal AF.

## Introduction

Atrial fibrillation (AF) is associated with left atrial (LA) remodeling and abnormal pulmonary vascular–right ventricular (RV) coupling due to the larger LA volume and myocardial fibrosis in patients with AF than in those with sinus rhythm (1, 2). LA enlargement is a risk factor for AF and stroke, while myocardial fibrosis due to LA remodeling causes electromechanical conduction delay and forms the substrate for AF (3, 4). It is also known that patients with persistent AF may have low treatment responsiveness or less reversible cardiac remodeling, which may affect cardiac function (3).

The risk of progression to permanent AF is greater in patients with larger reductions in LA compliance and strain (2). AF and RV dysfunction are common in patients with heart failure with preserved ejection fraction (HFpEF) and are known to be associated with poor prognosis when they occur. The pulmonary veins (PVs) are the main source of AF triggers, and radiofrequency catheter ablation (RFCA) is the most commonly performed and most effective treatment for patients with antiarrhythmic drug-refractory AF or symptoms (5). Several studies have shown that RV remodeling, which is associated with pulmonary artery systolic pressure (PAP), triggers LA remodeling and that LA volume is a more important predictor of recurrence after PV isolation (PVI) than the type of AF (3). Tricuspid regurgitation (TR), required when calculating PAP, is also well known to be associated with increased mortality regardless of patients with and without AF (6, 7).

PAP is a parameter reflecting cardiac remodeling that can be measured using echocardiography. In this study, we aimed to investigate whether PAP is associated with the recurrence of AF after RFCA.

## Methods

### Study population and design

All consecutive patients who underwent RFCA of AF between January 2005 and April 2019 were retrospectively analyzed. The Korea University Anam Hospital Institutional Review Board (No. 2021AN0018) approved the study protocol and waived the documentation of informed consent for this study.

### Echocardiography

**Figure 1:**
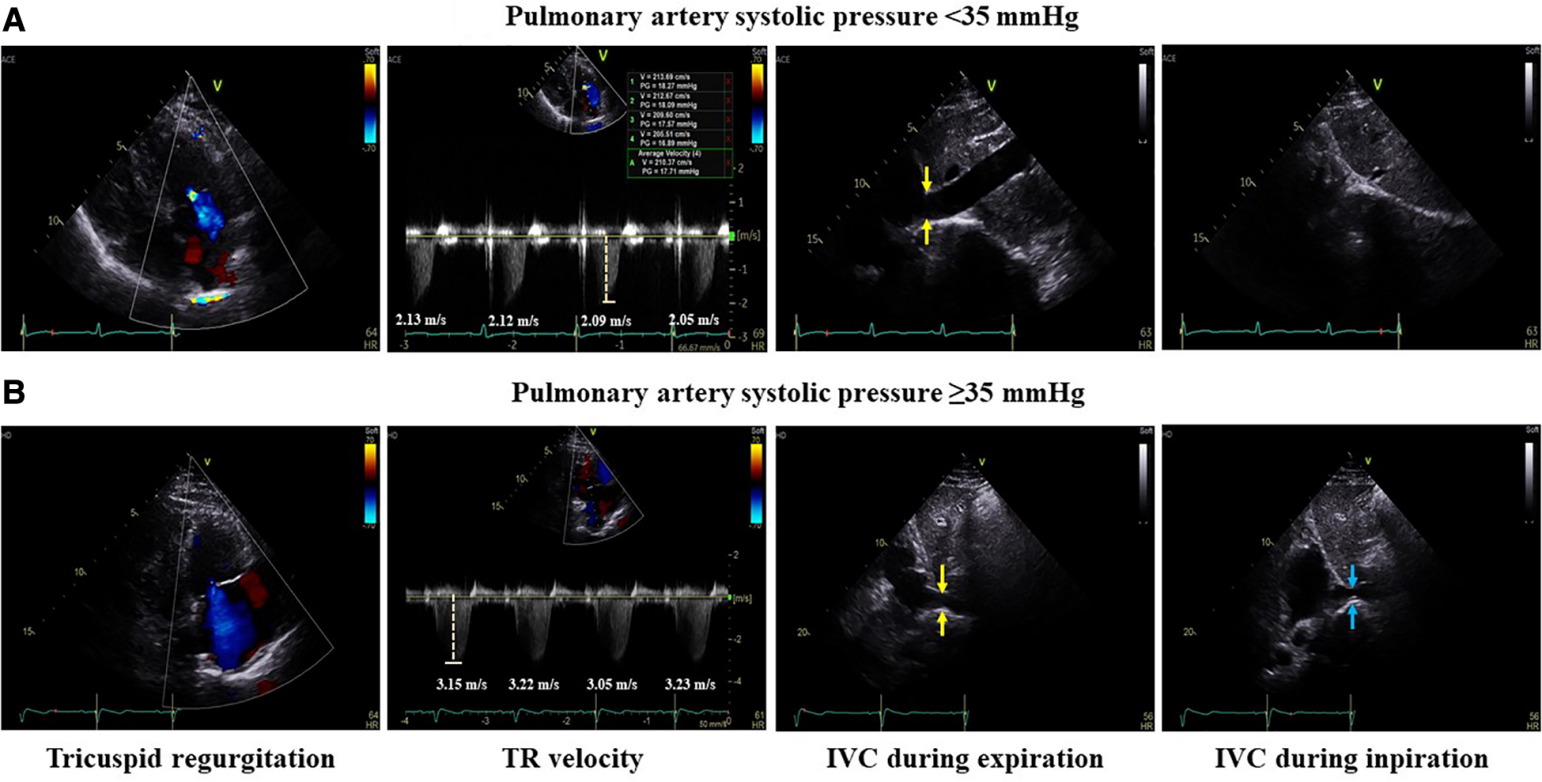
Measurement of pulmonary artery systolic pressure. Example of measurements of pulmonary artery systolic pressure <35 and ≥35 mmHg. The mean tricuspid regurgitation velocity was estimated by measuring the TR velocity of four or more beats. To evaluate the estimation of pulmonary pressure, right atrial pressure was assessed by measuring the diameter of the inferior vena cava (yellow arrow) and the respiratory collapse during inspiration (blue arrow). Pulmonary artery systolic pressure was estimated using the following formula. (**A**) In the case of PAP <35 mmHg, the mean TR velocity was 2.1 m/s and IVC almost completely collapsed during inspiration. So, pulmonary artery systolic pressure was 22.6 mmHg. (**B**) In the case of PAP ≥35 mmHg, the mean TR velocity was 3.2 m/s and IVC collapsed to less than 50% during inspiration. So, pulmonary artery systolic pressure was 55.9 mmHg. TR, tricuspid regurgitation; IVC, inferior vena cava; PAP, pulmonary artery pressure.

All patients underwent transthoracic echocardiography (TTE) (GE Vivid E9; Vingmed Ultrasound, Horten, Norway) before RFCA, and echocardiographic parameters were analyzed. The LA size was measured using two-dimensional-guided M-mode echocardiography according to the 2015 American Society of Echocardiography guidelines. The anteroposterior diameter of the left atrium was measured from the parasternal long-axis view at the end of the left ventricular (LV) systole (8). The normal values of LA anteroposterior diameter are 27–38 mm in women and 30–40 mm in men. Global LV function was assessed by measuring the difference between the end-diastolic and end-systolic estimates as follows: EF = ([end-diastolic volume]−[end-systolic volume])/[end-diastolic volume]. Pulmonary artery systolic pressure was estimated from the tricuspid regurgitation (TR) velocity. To evaluate the estimation of pulmonary pressure, right atrial (RA) pressure was assessed by measuring the diameter of the inferior vena cava and the respiratory collapse. Systolic PAP was estimated using the following formula: 4*TR peak velocity^2^ + RA pressure (Bernoulli equation)^4^ (Figure 1). Echocardiographic guidelines recommend measuring at least five beats in AF to assess average TR velocity (9). The TR velocity was measured once during SR to accurately measure systolic PAP, whereas the TR velocity was measured three times during AF to estimate the mean rate. All echocardiographic examinations were performed by a skilled echocardiographer with more than 3 years of experience and confirmed by a board-certified cardiologist.

### Electrophysiologic studies and catheter ablation

Patients were sedated by intravenous injection of midazolam and propofol, and arterial blood gas analysis was performed hourly. Ablation was performed using RFCA under three-dimensional (3D) electroanatomical mapping (CARTO3, Johnson & Johnson Inc., Diamond Bar, CA, USA; NavX, St. Jude Medical Inc., Minnetonka, MN, USA) merged with cardiac magnetic resonance imaging or 3D spiral computed tomography. Low RA and intracardiac electrocardiograms of the coronary sinus were mapped with a duodecapolar catheter (St. Jude Medical, Inc., Minnetonka, MN, USA), a decapolar catheter (Bard Electrophysiology, Inc., Lowell, MA, USA) was positioned in the high RA, and a quadripolar catheter was positioned in the right ventricle (RV). Two transseptal punctures were performed using long sheaths (Fast-Cath™ and Swartz™ SL1; St. Jude Medical Inc., AF Division, Minnetonka, MN, USA) and a Brockenbrough needle under fluoroscopic guidance. From the time of performing the septal puncture, unfractionated heparin was injected to maintain an activated clotting time between 300 and 350 s. Radiofrequency energy was delivered using a 3.5-mm open, irrigated-tip ablation catheter at a maximum temperature of 35°C and power of 25–35 W at the physicians' discretion. All patients underwent circumferential PVI. The ablation strategy was performed as previously described (10). We evaluated the triggers of sustained AF during catheter ablation for AF and targeted it after circumferential PVI. Multiple electrical cardioversions were performed in patients with AF initiated under infusion of isoproterenol (10–20 μg/min) to identify triggers that sustained AF. The endpoint of the procedure for patients with AF was to eliminate all triggers, including those of PVs. For patients with persistent AF, 3D-guided substrate modification or linear ablation was performed, including the roof line, posterior inferior line, peri-mitral isthmus line, or anterior line at the physicians' discretion. When AF was converted to atrial tachycardia (AT) during PVI or substrate modification, activation, and entrainment mapping were performed again, and additional ablation was performed. Cavotricuspid isthmus linear ablation was performed in patients with clinical atrial flutter, and the bidirectional block was confirmed.

### Follow-up and definition of recurrence after catheter ablation

If there were no complications, the patients were discharged within 2 days after RFCA and visited an outpatient clinic 2 weeks later. All patients had regular follow-up outpatient visits at 1, 3, 6, and every 6 months after that. Electrocardiography (ECG) was routinely performed at each outpatient visit, and 24-h Holter monitoring was performed at 3, 6, and 12 months after RFCA. If the patient had palpitations or dizziness, 24-h or 3-day Holter monitoring or event recording was performed to evaluate the cause. Atrial arrhythmias that lasted for more than 30 s within 3 months after the procedure were defined as early recurrences as a blanking period, and arrhythmias occurring 3 months later were defined as late recurrence. Antiarrhythmic drugs were taken for 3 months after the procedure and then discontinued at the physician's discretion.

### Statistical analysis

Continuous variables are presented as mean ± standard deviation. The Student's *t*-test was used to compare continuous variables between two groups of patients. Categorical variables were compared using the chi-square or Fisher's exact test. A Kaplan–Meier analysis using log-rank testing was performed to compare recurrence free rates. Univariate and multivariate Cox regression analyses were performed. Two-tailed *p*-values were reported, and results were considered statistically significant at *p *< 0.05. Statistical analyses were performed using SPSS statistical software (version 24.0; SPSS Inc., Chicago, IL, USA).

## Results

### Study patients

**Figure 2:**
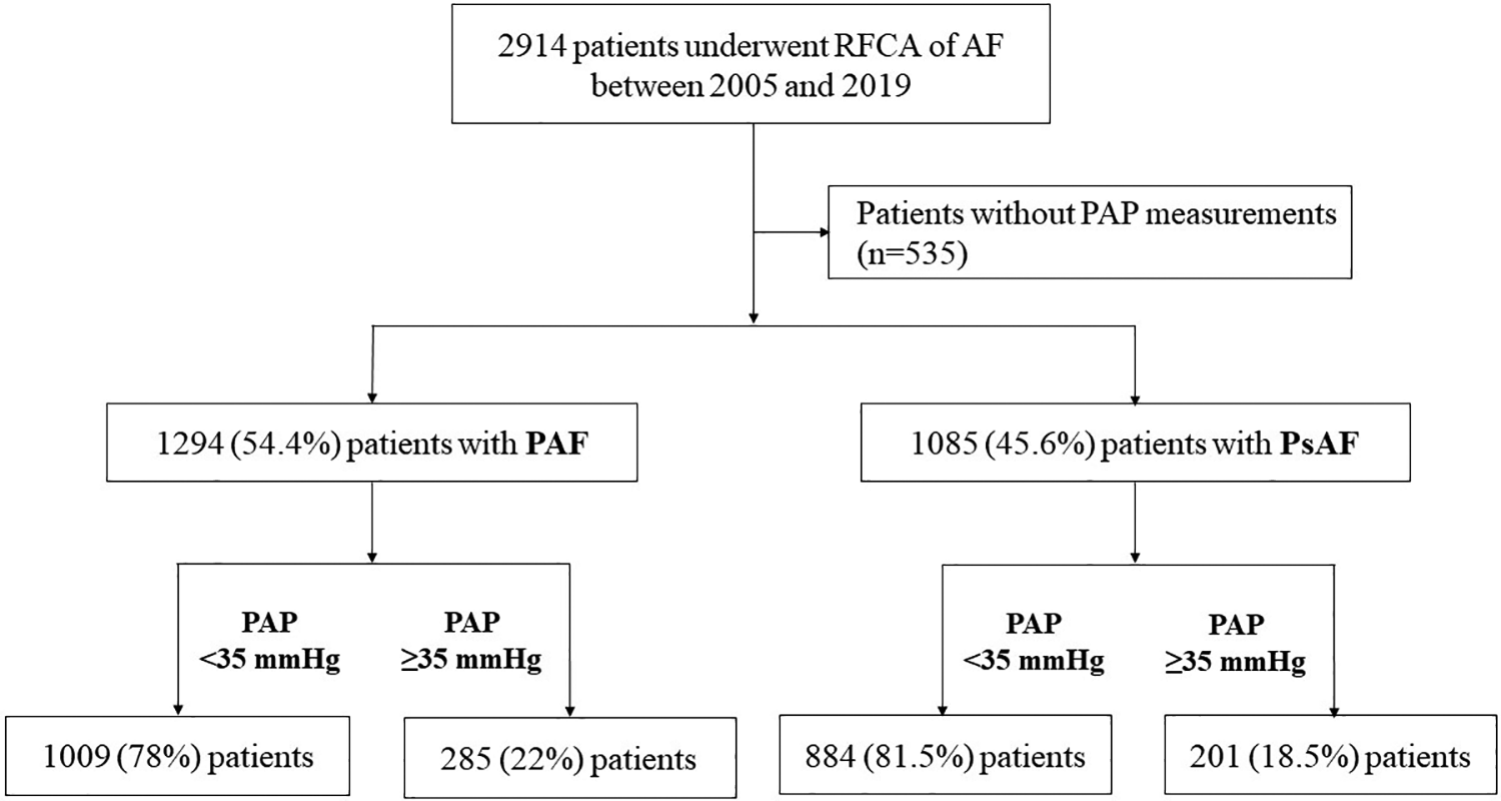
Flow of the study design. Among 2,379 consecutive patients who underwent RFCA for AF, 1,294 (54.4%) had PAF. According to the stratification based on PAP, 78% of patients with PAF had a PAP <35 mmHg. RFCA, radiofrequency catheter ablation; AF, atrial fibrillation; PAP, pulmonary artery pressure; PAF, paroxysmal atrial fibrillation; PsAF, persistent atrial fibrillation.

Among 2,914 consecutive patients, 535 patients without PAP measurements of which an adequate tricuspid regurgitation rate could not be estimated due to poor technical quality by TTE were excluded. The median follow-up duration was 25.4 months. Of the remaining 2,379 patients, 1,294 (54.4%) had paroxysmal AF and 1,085 (45.6%) had persistent AF. According to the stratification based on PAP, 22% of patients with paroxysmal AF and 18.5% of those with persistent AF had PAP ≥35 mmHg (Figure 2).

### Baseline characteristics

**Figure 3:**
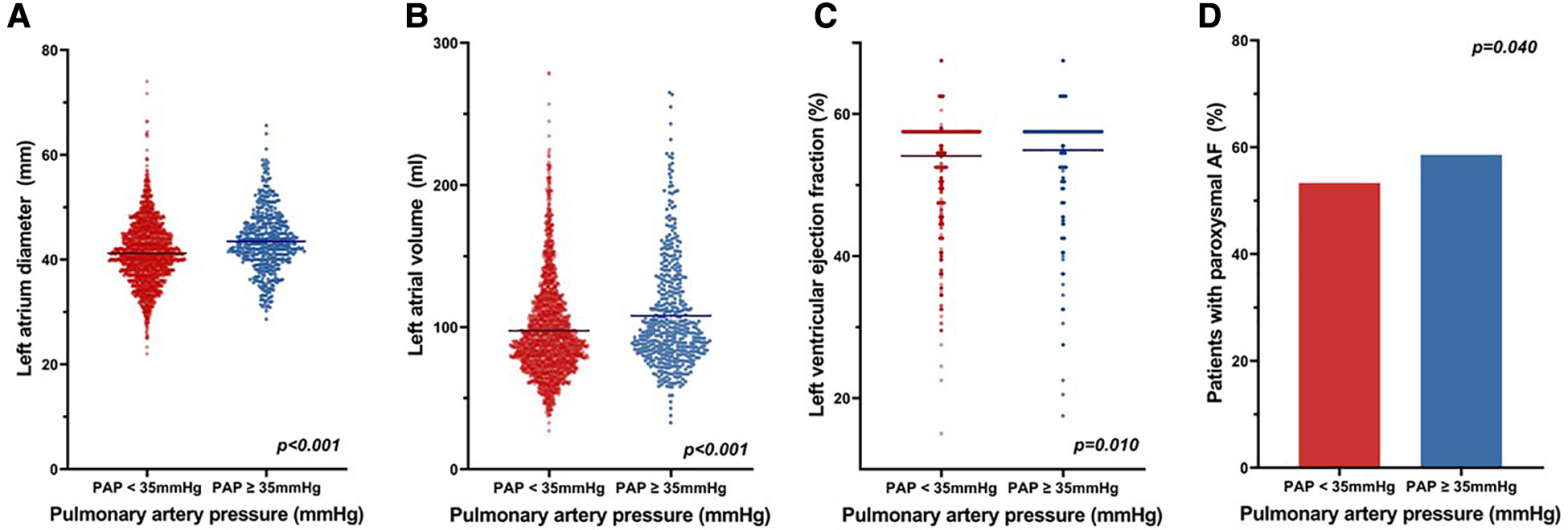
Scatter plot of the relation between PAP and LA. The scatter plot shows the relationship between PAP and (**A**) LA diameter, (**B**) LA volume, and (**C**) LVEF. (**D**) Bar graph shows that patients with PAP ≥35 mmHg were more likely to have paroxysmal AF. PAP, pulmonary artery pressure; LA, left atrial; AF, atrial fibrillation.

The baseline demographics of patients in the two groups are shown in Table 1. Patients with PAP <35 mmHg were younger and less likely to have a history of paroxysmal AF, hypertension, diabetes mellitus, or vascular disease and had a significantly lower median CHA_2_DS_2_-VASc (congestive heart failure, hypertension, age ≥75 years, diabetes, stroke, vascular disease, age 65–74 years, and female sex) score, E/e′, LA volume, and mean LA diameter than those with PAP ≥35 mmHg. LA pressure was measured in 34 patients with PAP ≥35 mmHg and 218 patients with PAP <35 mmHg. LA pressure was significantly higher in patients with PAP ≥35 mmHg than in patients with PAP <35 mmHg (17.7 ± 4.2 mmHg vs. 15.1 ± 4.8 mmHg, *p *= 0.003). Figure 3 shows a scatter plot of the relationship between PAP and LA diameter, LA volume, and LVEF. The bar graph shows that patients with PAP ≥35 mmHg were more likely to have paroxysmal AF. When comparing patients according to AF type, those with persistent AF had lower LVEF (52.6 ± 7.1% vs. 55.7 ± 4.9%, *p* < 0.001), larger LA volume (114.7 ± 38.7 mL vs. 86.8 ± 25.1 mL, *p* < 0.001), and greater LA enlargement (44.4 ± 5.5 mm vs. 39.4 ± 5.2 mm, *p* < 0.001) than those with paroxysmal AF. Substrate modification (56% vs. 9%, *p* < 0.001) and LA linear ablation (54% vs. 17.6%, *p* < 0.001) were significantly more frequently performed in persistent AF (Supplementary Table S1). When a non-PV trigger was confirmed during isoproterenol infusion, the non-PV trigger was eliminated. Among patients with paroxysmal AF, the rate of substrate modification was significantly different between the PAP <35 mmHg group and the PAP ≥35 mmHg group (8.6% vs. 10.2%, *p *= 0.07). However, among patients with persistent AF, substrate modification was more frequently performed in those with PAP <35 mmHg than in those with PAP ≥35 mmHg (57.9% vs. 47.3%, *p *= 0.07). The rate of LA linear ablation was not significantly different between the PAP <35 mmHg group and the PAP ≥35 mmHg group, regardless of the AF type (Supplementary Table S2).

**Table 1:** Baseline characteristics of patients who underwent AF ablation stratified according to PAP.

Variables	Total	PAP <35 mmHg	PAP ≥35 mmHg	*p*-Value
	(*n* = 2,379)	(*n* = 1,893)	(*n* = 486)	
Age (years)	56.7 ± 10.6	55.8 ± 10.5	60.3 ± 10.3	<0.001
Male, *n* (%)	1,831 (77)	1,512 (79.9)	319 (65.6)	<0.001
Bodyweight (kg)	70.5 ± 11.2	71 ± 10.9	68.3 ± 12.1	<0.001
Height (cm)	167.9 ± 8.4	168.6 ± 8.2	165.2 ± 8.6	<0.001
BMI (kg/m^2^)	24.9 ± 3.0	24.9 ± 2.9	24.9 ± 3.3	0.900
Comorbidities				
Paroxysmal AF, *n* (%)	1,294 (54.4)	1,009 (53.3)	285 (58.6)	0.040
Congestive HF, *n* (%)	162 (6.8)	129 (6.8)	33 (6.9)	0.900
Hypertension, *n* (%)	913 (38.4)	691 (36.5)	222 (45.7)	<0.001
Diabetes mellitus, *n* (%)	220 (9.2)	157 (8.3)	63 (12.7)	0.002
Previous stroke, *n* (%)	212 (8.9)	161 (8.5)	51 (10.5)	0.170
Vascular disease, *n* (%)	187 (7.9)	135 (7.1)	52 (10.7)	0.009
CHA_2_DS_2_-VASc score	1.3 ± 1.3	1.17 ± 1.2	1.71 ± 1.3	<0.001
Echocardiographic characteristics				
LVEF (%)	54.3 ± 6.2	54.1 ± 6.1	54.9 ± 6.5	0.010
Left atrium diameter (mm)	39.3 ± 5.2	41.2 ± 5.9	43.4 ± 5.9	<0.001
Left atrial volume (mL)	99.5 ± 34.9	97.4 ± 33.5	107.8 ± 38.8	<0.001
E/e′	9.0 ± 4.3	8.6 ± 3.7	10.5 ± 5.7	<0.001
Ablation, *n* (%)				
Substrate modification	725 (30.5)	600 (31.7)	125 (25.7)	0.011
LA Linear ablation	814 (34.2)	649 (34.3)	165 (34.0)	0.915
CTI	1,695 (71.2)	1,349 (71.3)	346 (71.2)	0.976
SVC isolation	186 (7.8)	152 (8.0)	34 (7.0)	0.507
LA pressure during procedure^a^	15.4 ± 4.8	15.1 ± 4.8	17.7 ± 4.2	0.003

PAP, pulmonary artery pressure; BMI, body mass index; CHA_2_DS_2_-VASc, AF, atrial fibrillation; congestive heart failure, hypertension, age ≥75 years, diabetes, stroke, vascular disease, age 65–74 years, and female sex; HF, heart failure; LVEF, left ventricular ejection fraction; LA, left atrium; CTI, cavotricuspid isthmus; SVC, superior vena cava.

^a^ A total of 218 patients with PAP <35 mmHg and 34 patients with PAP ≥35 mmHg were performed LA pressure during the procedure.

### Recurrence of AF after RFCA

**Figure 4:**
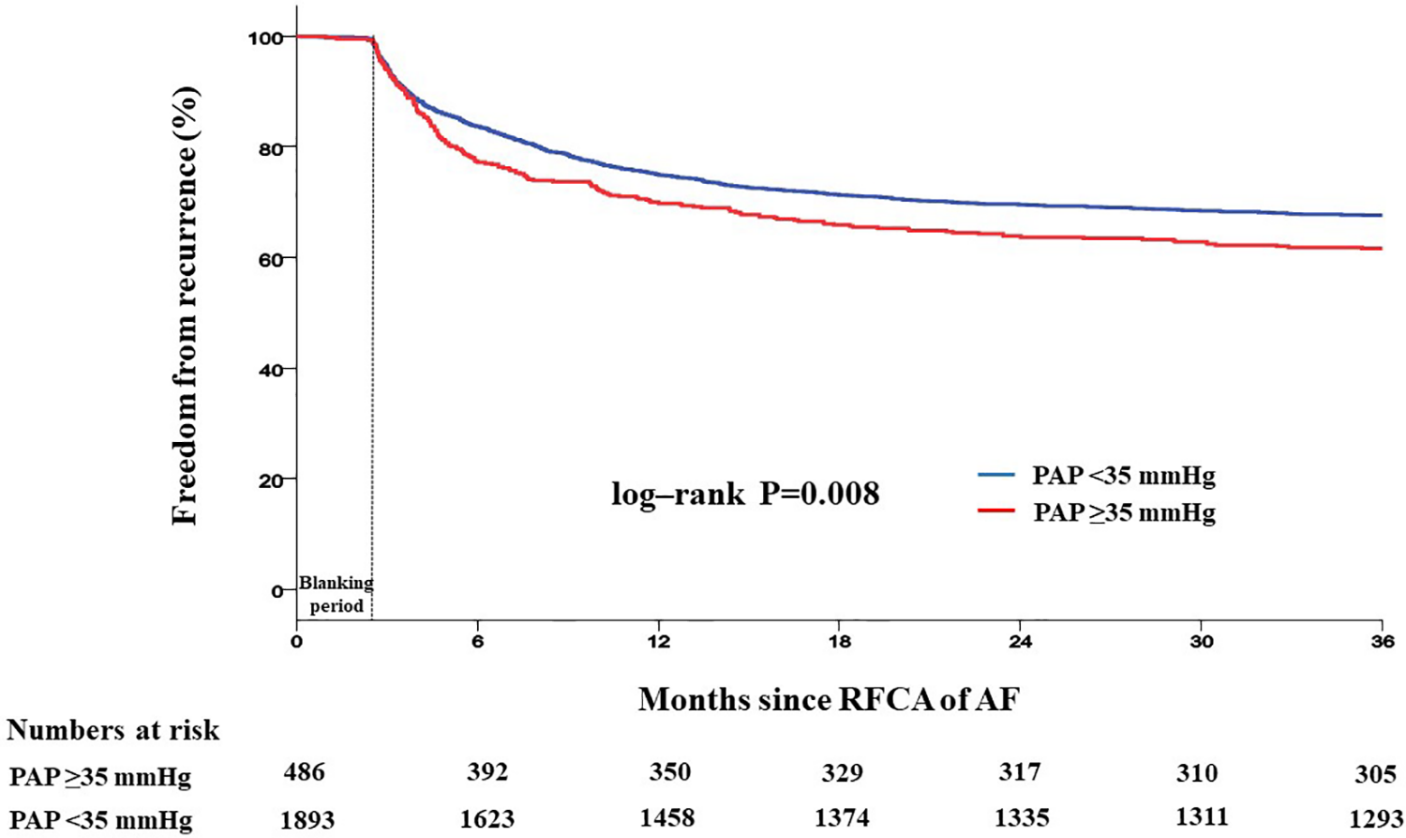
Kaplan–Meier analysis of freedom from recurrence according to PAP. Kaplan–Meier analysis with log-rank test was performed to compare freedom from recurrence. Patients with PAP ≥35 mmHg showed a significantly higher recurrence rate than those with PAP <35 mmHg. RFCA, radiofrequency catheter ablation; AF, atrial fibrillation; PAP, pulmonary artery pressure.

**Figure 5:**
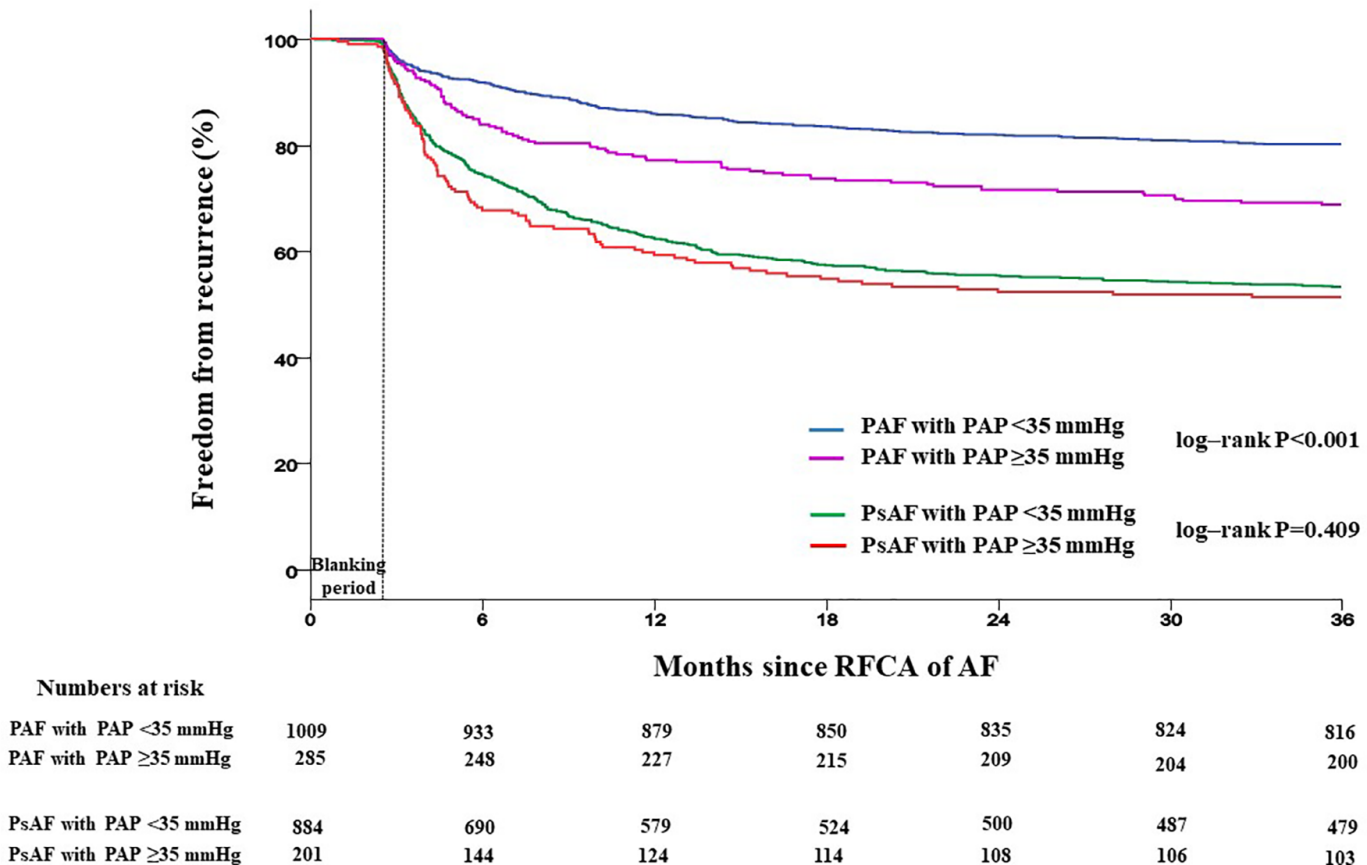
Kaplan–Meier analysis of freedom from recurrence according to AF type. Kaplan–Meier analysis with log-rank test was performed to compare freedom from recurrence rate. Among patients with paroxysmal AF, the recurrence rate was significantly higher in those with PAP ≥35 mmHg than in those with PAP <35 mmHg. However, among patients with persistent AF, the recurrence rate was not significantly different between the two groups. RFCA, radiofrequency catheter ablation; AF, atrial fibrillation; PAP, pulmonary artery pressure; PAF, paroxysmal atrial fibrillation; PsAF, persistent atrial fibrillation.

Patients with PAP ≥35 mmHg showed a significantly higher recurrence rate than those with PAP <35 mmHg (42.2% vs. 35.9%, log-rank *p *= 0.008) (Figure 4). Recurrence occurred as AF in 547 (61.9%) patients and as AT in 337 (38.1%) patients. Among patients with PAP <35 mmHg, recurrence occurred as AF in 60.8% and as organized AT in 39.2%, whereas in those with PAP ≥35 mmHg, recurrence occurred as AF and AT in 65.2% and 34.6%, respectively (Supplementary Figure S1). When comparing the rate of recurrence according to AF type, 340 (26.3%) were in paroxysmal AF and 544 (50.1%) were in persistent AF (Supplementary Figure S2). Among patients with paroxysmal AF, the recurrence rate was significantly higher in those with PAP ≥35 mmHg than in those with PAP <35 mmHg (35.1% vs. 23.8%, log-rank *p *= 0.008). However, among patients with persistent AF, the recurrence rate was not significantly different between the two groups (49.7% vs. 52.2%, log-rank *p *= 0.409) (Figure 5).

### PAP predicts recurrence

The results of Cox regression analysis for AF recurrence are presented in Table 2. In univariate analysis, PAP ≥35 mmHg [hazard ratio (HR) 1.17, 95% confidence interval (CI) 1.00–1.37, *p *= 0.046], history of congestive heart failure (HR 1.40, 95% CI 1.10–1.78, *p *= 0.007), and stroke/TIA (HR 1.35, 95% CI 1.08–1.67, *p *= 0.007) were associated with an increased risk of recurrence of atrial tachyarrhythmias. Substrate modification (HR 2.55, 95% CI 2.23–2.91, *p *= 0.001) and LA linear ablation (HR 1.48, 95% CI 1.30–1.69, *p *= 0.001) were also associated with an increased risk of recurrence. Multivariate analysis showed that PAP ≥35 mmHg was independently associated with recurrence (HR 1.19, 95% CI 1.02–1.40, *p *= 0.027) after adjusting for age, sex, congestive heart failure, stroke/TIA, PAP, LVEF, substrate modification, and linear ablation.

**Table 2:** Cox regression analysis for recurrence of atrial arrhythmia after RFCA.

	Univariate		Multivariate	
	HR (95% CI)	*p*-Value	HR (95% Cl)	*p*-Value
Age ≥65 (years)	1.05 (0.90–1.22)	0.528		
Male sex	1.12 (0.95–1.31)	0.166		
Congestive heart failure	1.40 (1.10–1.78)	0.007		
Hypertension	1.04 (0.91–1.19)	0.534		
Diabetes mellitus	1.14 (0.92–1.41)	0.244		
Stroke/TIA	1.35 (1.08–1.67)	0.007	1.28 (1.23–1.59)	0.027
Vascular disease	1.12 (0.90–1.40)	0.303		
PAP ≥35 (mmHg)	1.17 (1.00–1.37)	0.046	1.19 (1.02–1.40)	0.027
LA diameter ≥40 (mm)	0.77 (0.29–2.06)	0.603		
LV EF <40 (%)	1.34 (0.98–1.83)	0.067		
E/e′	1.01 (0.99–1.02)	0.275		
Substrate modification	2.55 (2.23–2.91)	<0.001	2.97 (2.48–3.56)	<0.001
LA linear ablation	1.48 (1.30–1.69)	<0.001		

Factors significant in univariate analysis (*P* < 0.05) were entered into multivariate analysis using Cox regression and adjusted for age, sex, PAP, LVEF, congestive heart failure, Stroke/TIA, substrate modification, and LA linear ablation.

RFCA, radiofrequency catheter ablation; HR, hazard ratio; CI, confidence interval; TIA, transient ischemic attack; PAP, pulmonary artery pressure; LA, left atrium; LVEF, left ventricular ejection fraction.

## Discussion

The major findings of this study are as follows: First, patients with PAP ≥35 mmHg showed a significantly higher recurrence rate than those with PAP <35 mmHg. Second, among patients with paroxysmal AF, the recurrence rate was higher in those with PAP ≥35 mmHg than in those with PAP <35 mmHg; however, in patients with persistent AF, the recurrence rate was not significantly different between the two groups. Third, PAP ≥35 mmHg was independently associated with recurrence (Supplementary Figure S3).

RFCA of AF has been demonstrated to improve quality of life better than administering antiarrhythmic drugs. RFCA reduces the risk of stroke in patients with AF and improves all-cause mortality in patients with both AF and heart failure, according to the CASTLE-AF trial (11, 12). However, despite recent data on reduced recurrence after RFCA, the recurrence of atrial arrhythmia remains challenging. Several studies have shown that a history of diabetes mellitus, hypertension, impaired renal function, increasing CHA_2_DS_2_-VASc score, and long duration of AF significantly increase the risk of AF recurrence (13, 14). Also, among echocardiographic risk factors, enlarged LA diameter, decreased ejection fraction, and increased E/e′ are known to be associated with increased late recurrence (12). We found that PAP ≥35 mmHg was a robust risk factor that could predict AF recurrence after RFCA.

### LA remodeling and AF

LA volume is an important predictor of recurrence after RFCA (4), and our study also found that an LA volume of ≥80 mL was associated with an increased risk of recurrence. A larger LA volume increases the LA surface area, wall thickness, and stress. This is associated with the possibility of PV reconduction because of the formation of a more transmural lesion (15). Therefore, the larger the LA volume, the greater the likelihood of reconduction, which may be a predictor of recurrence. LVEF <50% has also been shown to be associated with recurrence after RFCA. LV dysfunction can increase the RV size, LA filling pressure, and LA volume. The increasing burden of AF during pathophysiologic progression has been associated with decreased LA reservoir strain and reduced LA compliance, induced by abnormal LA mechanics, remodeling, and RV–pulmonary artery coupling (2). Among patients with HFpEF, those with AF have a twofold larger atrial volume, a greater burden of RV dysfunction, and poorer exercise capacity than those without AF (15, 16). Therefore, as the burden of AF increases, the LA size increases. Roh et al. demonstrated the association between late gadolinium enhancement (LGE), which indicates fibrosis on MRI, and LA pressure, showing that the recurrence rate in patients with persistent AF was significantly higher in the extent-LGE group (LGE ≥20%) than in the small-LGE group (LGE <20%) (17). In our study, the recurrence rate was significantly different according to PAP in patients with paroxysmal AF before LA remodeling, and LGE was significantly larger in patients with persistent AF compared to those with paroxysmal AF (17.2 ± 10.6 vs. 15.4 ± 10.3%, *p *= 0.005) (Supplementary Table S1), indicating that fibrosis was more advanced in persistent AF. Therefore, PAP measured by TTE, a noninvasive method, is effective in predicting recurrence in patients with paroxysmal AF.

### Pulmonary vascular impairment and AF

The left atrium is known to play an important role in the occurrence of AF. However, AF can also develop in patients with right-sided heart disease (18). Atrial remodeling, including fibrosis, is common in patients with AF (19). In a previous study, monocrotaline rats show a fivefold higher RA fibrous tissue content than control rats. Atrial fibrosis is a major cause of conduction changes in AF, and the RA mRNA expression levels of fibrosis-related genes *FnI*, *Col_1α1_*_,_ *Col_3α1_*_,_ *Mmp2*, *Mmp*, and *Acta_1_* were upregulated in MCT rats (20). Patients with pulmonary hypertension (PH) show reduced electrogram voltage, slower RA conduction, and electrically silent areas due to fibrous scarring and increased AF induction (21). In patients with HFpEF and AF, the cardiac volume increases as the AF burden increases, which has been demonstrated to be due to higher RA-to-pulmonary capillary wedge pressure (PCWP) ratio and RA pressure (2). Kawasaki et al. suggested that LA pressure, rather than LA function or volume, may be an important risk factor for the recurrence of AF after ablation (22).

The E/e′ ratio has been suggested as an initial measurement to estimate LV filling pressure, especially in patients with preserved systolic function. Elevated LA pressure lowers pulmonary artery compliance. Therefore, elevated LV filling pressure increases the RV afterload and decreases pulmonary artery compliance (23).

### Catheter ablation and PAP

Among patients with paroxysmal AF, substrate modification was more frequently performed among those with PAP ≥35 mmHg. That is why increasing PAP may affect the LA pressure and LA volume, which can potentially lead to more AF triggers and recurrences. Conversely, among patients with persistent AF, additional ablation, such as substrate modification or line ablation, was more frequently performed among those with PAP <35 mmHg. This may be caused by an existing LA in persistent AF, and more remodeling occurs when PAP is ≥35 mmHg (Supplementary Table S2).

### Limitations

This study had several limitations. First, right heart catheterization or LA pressure assessment before ablation was not performed simultaneously with echocardiography; therefore, LA pressure data were obtained from 252 patients. Second, the measured values varied depending on the presence of AF or sinus rhythm during the measurements of echocardiographic parameters, especially LVEF and E/e′ ratio. However, our hospital has a standard protocol for measuring TR velocity during AF. Third, the etiology of TR, such as functional TR or isolated TR, has not been classified. Fourth, there were limited data on MRI, inflammatory markers, brain natriuretic peptide, and history of lung disease that identify fibrosis. More analyses about the markers in the future are warranted to better assess the causal link between AF recurrence postablation and the augmentation of PAP.

### Clinical implications

In this study, most patients with AF and PAP ≥35 mmHg had EF >50%, E/e′ ratio <15, and PCWP <20 mmHg. Thus, PH is more likely due to an increase in LA pressure. The E/e′ ratio is correlated with PCWP (24). Therefore, it can be presumed that PH is less likely to be caused by congestive heart failure. This can explain the strong association between elevated PAP and increased LA pressure, subsequently increasing the risk of AF recurrence. As an indirect measure of LA pressure, echocardiography is a useful noninvasive method.

## Conclusion

This study demonstrated that a higher PAP was associated with an increased risk of recurrence after RFCA in patients with paroxysmal AF, suggesting a mechanism by which PAP may influence the remodeling process at the junction between the left atrium and PV. This study also showed that PAP ≥35 mmHg was an independent factor for atrial arrhythmia recurrence. Therefore, RFCA should be considered before the remodeling process proceeds by measuring PAP.

## Data Availability

The raw data supporting the conclusions of this article will be made available by the authors without undue reservation.
