# Supplementary material for: Impact of pulmonary artery pressure on recurrence after catheter ablation in patients with atrial fibrillation
Source: Front Cardiovasc Med. 2023 Sep 4;10:1187774. doi: 10.3389/fcvm.2023.1187774 (PMC10507172; doi:10.3389/fcvm.2023.1187774)
Supplement: Supplementary file 1 [file Datasheet1.pdf]

Supplementary Table S1. Baseline characteristics of patients stratified according to AF type

Variables	Paroxysmal AF (n=1294)	Persistent AF (n=1085)	*p*-Value
Age, (years)	56.56±11.1	56.9±9.9	0.426
Male, n (%)	940 (72.6)	891 (82.1)	<0.001
Body weight (kg)	68.721±11	72.534±11	<0.001
Height (cm)	167.26 ± 8.5	168.65±8.2	<0.001
BMI (kg/m2)	24.49±2.9	25.45±3.1	<0.001
Congestive heart failure, n (%)	53 (4.0)	109 (10)	<0.001
Hypertension, n (%)	491 (38)	422 (39)	0.635
Diabetes, n (%)	113 (8.7)	107 (9.8)	0.344
Previous stroke, n (%)	97 (7.5)	115 (10.6)	0.008
Vascular disease, n (%)	101 (7.8)	86 (7.9)	0.913
CHA_2DS_2-VASc score	1.28±1.2	1.28±1.3	0.944
Pulmonary artery pressure (mmHg)	30.9±5.5	30.4±5.0	0.040
PAP ≥35 mmHg, n (%)	285 (22)	201 (18.5)	0.036
Left ventricular ejection fraction (%)	55.65±4.9	52.58±7.1	<0.001
Left atrial diameter (mm)	39.42±5.2	44.41±5.5	<0.001
Left atrial volume (ml)	86.8±25.1	114.7±38.7	<0.001
E/e’	9.0±4.2	8.9±4.4	0.638
Late gadolinium enhancement (%) *	15.4±10.3	17.2±10.6	0.005
Ablation, n (%)			
Substrate modification	117 (9.0)	608 (56.0)	<0.001
LA Linear ablation	228 (17.6)	586 (54.0)	<0.001
CTI	680 (52.6)	1015 (93.5)	<0.001
SVC isolation	115 (8.9)	71 (6.5)	0.038

*521 patients with paroxysmal AF and 462 patients with persistent AF was performed MRI

AF = atrial fibrillation; BMI = body mass index; CHA_2DS_2-VASc = congestive heart failure, hypertension, age ≥75 years, diabetes, stroke, vascular disease, age 65–74 years, and female sex; PAP = pulmonary artery pressure; LA = left atrium; CTI = cavotricuspid isthmus; SVC = superior vena cava; MRI = magnetic resonance imaging

Supplementary Table S2. Ablation for extra-PV potential stratified according to PAP

Variables	PAF (n=1294)			PsAF (n=1085)		
	PAP <35 mmHg (n=1009)	PAP ≥35 mmHg (n=285)	*p*-Value	PAP <35 mmHg (n=884)	PAP ≥35 mmHg (n=201)	*p*-Value
Substrate modification, n (%)	87 (8.6)	29 (10.2)	0.07	512 (57.9)	95 (47.3)	<0.01
LA linear ablation, n (%)	171 (16.9)	58 (20.4)	0.19	479 (54.2)	107 (53.2)	0.81
Roof line ablation, n (%)	96 (9.5)	35 (12.3)	0.03	392 (44.3)	87 (43.3)	0.06
Anterior line ablation, n (%)	67 (6.6)	26 (9.1)	0.04	294 (33.3)	59 (29.4)	0.04
Posterior line ablation, n (%)	29 (2.9)	7 (2.5)	0.16	103 (11.7)	25 (12.4)	0.09
Perimitral isolation, n (%)	81 (8.0)	24 (8.4)	0.09	193 (21.8)	52 (25.9)	0.03
CTI ablation, n (%)	520 (51.5)	159 (55.8)	0.02	829 (93.8)	186 (92.5)	0.10
SVC isolation, n (%)	95 (9.4)	20 (7.0)	0.04	56 (6.3)	14 (7.0)	0.12

PV = pulmonary vein; PAP = pulmonary artery pressure; CTI = cavotricuspid isthmus; SVC = superior vena cava

Supplementary Figure S1. Types of recurrence after RFCA

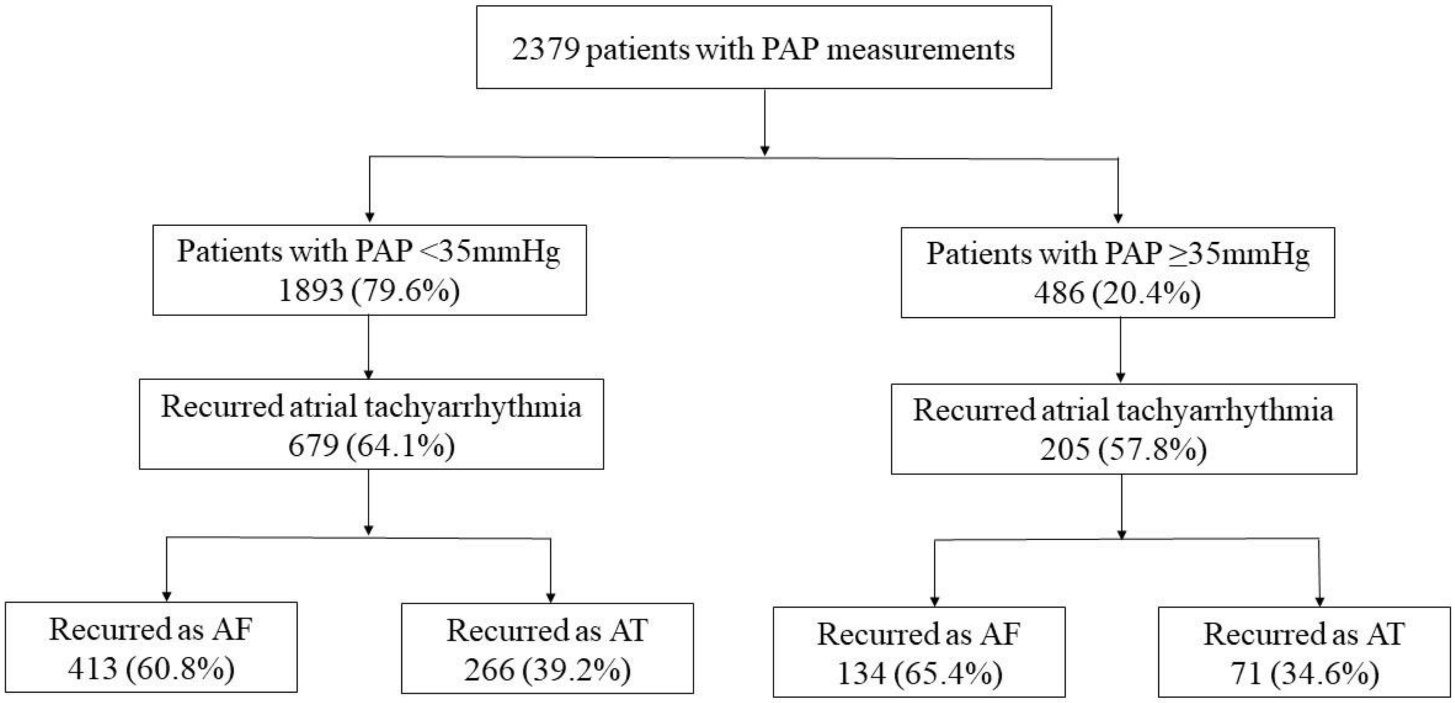

RFCA = radiofrequency catheter ablation; PAP = pulmonary artery pressure; AF = atrial fibrillation; AT = atrial tachycardia.

Supplementary Figure S2. Recurrent rate according to AF type

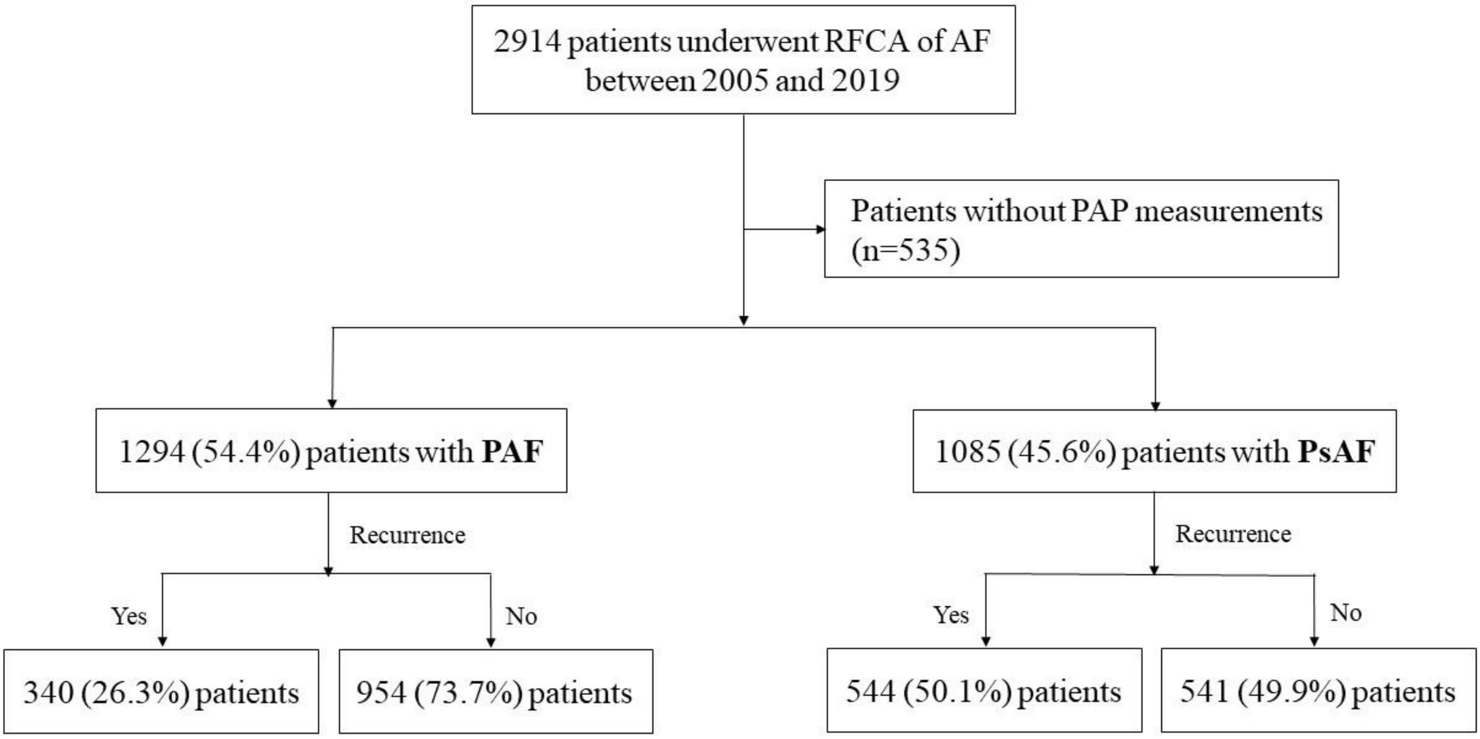

RFCA = radiofrequency catheter ablation; PAP = pulmonary artery pressure; AF = atrial fibrillation; PAF = paroxysmal atrial fibrillation; PsAF = persistent atrial fibrillation.

Supplementary Figure S3. Graphical abstract

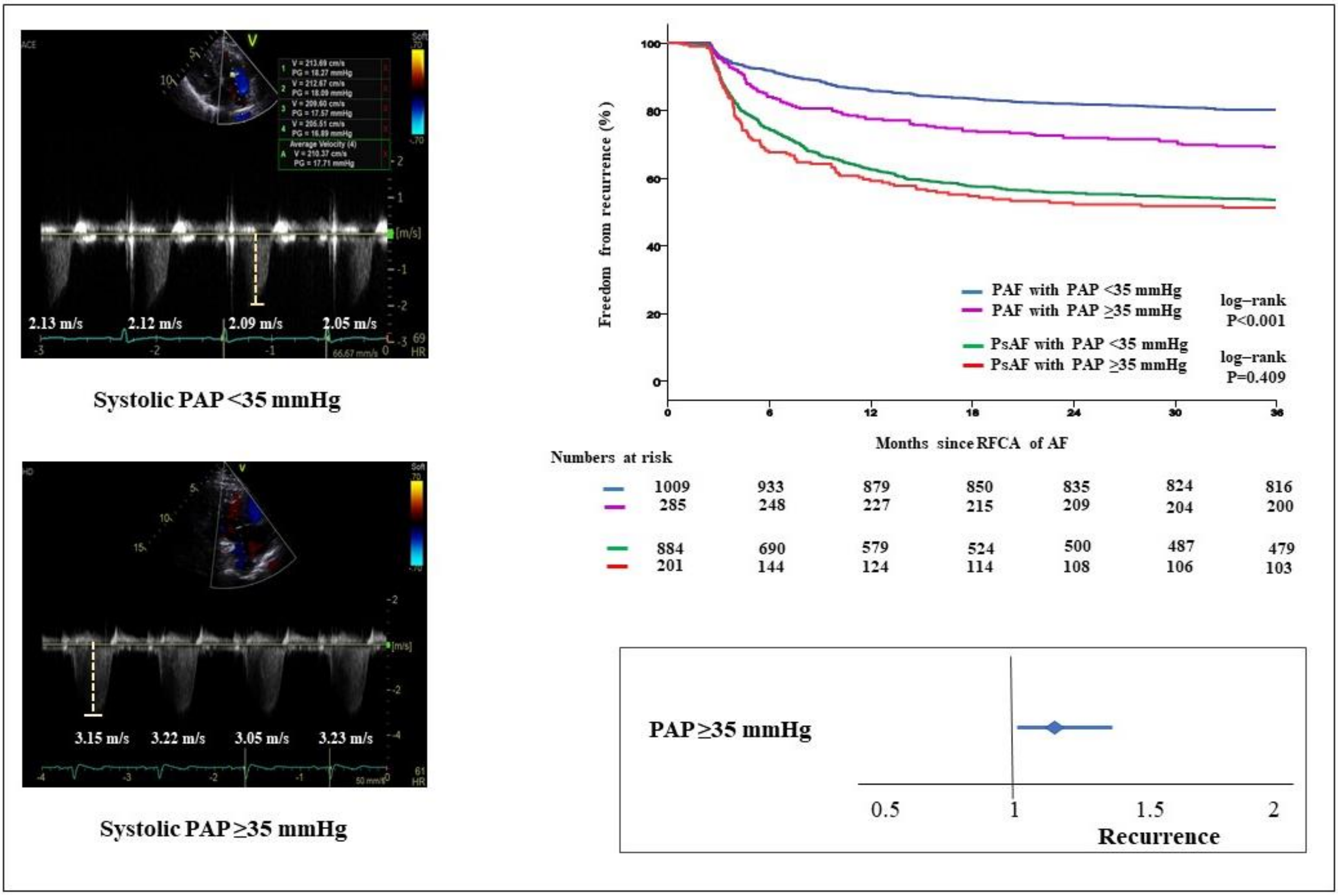

PAP = pulmonary artery pressure; PAF = paroxysmal atrial fibrillation; PsAF = persistent atrial fibrillation.
